# How TikTok Is Being Used to Help Individuals Cope With Breast Cancer: Cross-sectional Content Analysis

**DOI:** 10.2196/42245

**Published:** 2022-12-06

**Authors:** Corey H Basch, Grace C Hillyer, Bhavya Yalamanchili, Aldean Morris

**Affiliations:** 1 Department of Public Health William Paterson University Wayne, NJ United States; 2 Department of Epidemiology Mailman School of Public Health Columbia University New York, NY United States

**Keywords:** TikTok, breast cancer, social media, short video apps, social support, content analysis, video, patient support, medical information, health information, peer support, online conversation, online health information

## Abstract

**Background:**

Acknowledging the popularity of TikTok, how quickly medical information can spread, and how users seek support on social media, there is a clear lack of research on breast cancer conversations on TikTok. There is a paucity of information on how these videos can advocate for those impacted by breast cancer as a means to provide support and information as well as raise awareness.

**Objective:**

The purpose of this cross-sectional content analysis was to describe the content of videos from the hashtag #breastcancer on TikTok. Content related to breast cancer support and coping, cancer education, and heightening the awareness of breast cancer early detection, prevention, and treatment was evaluated.

**Methods:**

This study included 100 of the most viewed TikTok videos related to breast cancer through June 30, 2022. Videos were excluded if they were not in the English language or relevant to the topic being studied. Content was deductively coded into categories related to video characteristics and content topics using a screener based on expert breast cancer information sheets. Univariable analyses were conducted to evaluate differences in video characteristics and content when stratified as advocating or not advocating for breast cancer (yes or no) support, education, and awareness.

**Results:**

The cumulative number of views of the videos included in this study was 369,504,590. The majority (n=81, 81%) of videos were created by patients and loved ones of individuals with breast cancer, and the most commonly discussed topic was breast cancer support (n=88, 88%), followed by coping with the myriad issues surrounding breast cancer (n=79, 79%). Overall, <50% of the videos addressed important issues such as body image (n=48, 48%), surgery (n=46, 46%), medication and therapy (n=41, 41%), or the stigma associated with a breast cancer diagnosis (n=44, 44%); however, in videos that were advocacy oriented, body image (40/62, 64% vs 8/38, 21%; *P*<.001), stigma associated with breast cancer (33/62, 53% vs 11/38, 29%; *P*=.02), and breast cancer surgery (36/62, 58% vs 10/38, 26%; *P*=.002) were discussed significantly more often than in videos that did not specifically advocate for breast cancer.

**Conclusions:**

The use of videos to display health journeys can facilitate engagement by patients, family members, and loved ones interested in information about challenging conditions. Collectively, these findings highlight the level of peer-to-peer involvement on TikTok and may provide insights for designing breast cancer educational campaigns.

## Introduction

Globally, the World Health Organization reports that breast cancer is the most common newly diagnosed cancer [1], only just recently edging out lung cancer. There were 2.3 million cases of breast cancer diagnosed globally during 2020 and 685,000 breast cancer deaths [2]. The incidence of breast cancer is increasing in most countries [3], and it is the most prevalent in high-income countries [4]. The incidence of breast cancer, particularly in women aged ≥20 years from 2004 to 2018 [5], is believed to be due to controllable risk factors, which also increased during that time [5].

The American Cancer Society reports that breast cancer is the second most common cancer among women in the United States [6] and projects that there will be approximately 287,850 new cases of invasive breast cancer in women, with about 43,250 breast cancer deaths in the United States in 2022 [6]. Given the expansive impact of breast cancer, it is imperative that the general public become as informed as possible about breast cancer detection and treatment. Social media has become an incredibly popular mechanism for attaining such information.

In fact, according to a Pew Research Center survey of adults, 7 in 10 (72%) Americans use social media [7], and a similar percentage have sought out health information on the web [8]. People with chronic diseases such as cancer are most likely to seek out others with similar diagnoses through social media [8]. For example, Facebook, YouTube, Twitter, and Quora share an enormous, combined audience of 5 billion viewers, and studies on these platforms suggest that there is an active level of discourse related to breast cancer on them [9].

Further, there is more traffic on social media, specifically Twitter and Instagram, regarding women’s reproductive cancers than there is traffic about male reproductive cancers in relation to targeted campaigns [10]. Vraga et al [10] theorize that this finding is because breast cancer awareness campaigns have branded their cause well, with a pink ribbon and other pink symbols, and have also engaged powerful partners such as the National Football League to increase awareness.

Other social media platforms are also active on the subject of cancer [11-16]. An increase of over 300% in tweets associated with breast cancer was observed during Breast Cancer Awareness Month from 2012 to 2018 [17]. There is also a relationship between Twitter participation and improvements in patients’ self-reported knowledge about breast cancer [18]. Twitter generated more traffic when it came to both male and female reproductive cancer campaigns than Instagram, leading researchers to consider Instagram as an underused resource for the communication of information to the public about these cancers [10]. The most shared material on social networking sites is personal or social in nature; two-thirds of posts portray true experiences or otherwise provide support [19]. It is also used as a form of self-distraction from the stressors caused by a new, recurring, or terminal illness [20], which is helpful to both providers and patients.

Cancer advocacy works to improve the lives of people with cancer. Of the several key elements of cancer advocacy [21], social media platforms are the best positioned to guide individuals (eg, listening and sharing personal stories and providing support), educate about cancer, and raise awareness of important issues. Web-based peer-to-peer support can reduce social isolation and address unmet support needs by connecting individuals, especially younger individuals, to share their experiences and validate their treatment and life concerns [22].

TikTok is one of the most popular applications in 2022, attracting an audience of 1.5 billion active users [23]. The TikTok audience is generally younger, which could explain why TikTok has been underused in breast cancer awareness campaigns in the past. Further, breast cancer awareness campaigns are usually targeted at an older audience. However, a substantial proportion of young people are diagnosed with breast cancer, and it is beneficial to create awareness and discussion from an earlier age for early detection and treatment purposes. More than half (67%) of teens aged 13-17 years use TikTok, with 73% of girls aged 13-17 years using TikTok [24]. Acknowledging the popularity of TikTok, how quickly medical information can spread, and how users seek support on social media, there is a clear lack of research on breast cancer conversations on TikTok. Therefore, the purpose of this cross-sectional content analysis was to describe the content of videos from the hashtag #breastcancer on TikTok and to assess the breast cancer advocacy potential of these videos.

## Methods

### Data Collection

This study included the 100 most viewed TikTok videos related to breast cancer as a means of evaluating the content and messages seen by individuals at the time of data collection (through June 30, 2022). At the start of the study, the hashtag #breastcancer was the most popular, with 1.1 billion views; thus, our sample of 100 videos with 369,504,590 views represents approximately one-third of breast cancer–related Tik Tok videos viewed. Videos were excluded if they were not in the English language or relevant to the topic being studied. In total, 6 of the top 100 videos were not in English and 2 were not relevant to the study (8 in total). Thus, the next 8 most liked, relevant videos in English were included.

Data were collected by watching and analyzing the videos for mentions or suggestions of predetermined content categories. The content categories were created based on breast cancer and the breast cancer gene (fact sheets from the Mayo Clinic [25], a well-known and respected source of expert-vetted medical information). For each video, the link, date of posting, views, likes, comments, and shares were collected. The type of creator and content were also analyzed.

The content categories included the use of dance, music, or humor; mention or suggestion of cancer; new diagnosis; relapse or recurrent cancer; breast cancer gene testing; advocacy (ie, content that advocated for breast cancer support, provided breast cancer information to educate viewers, and raised awareness of breast cancer issues such as early detection, prevention, and treatment); body image; hair loss; anxiety; stigma; support; coping; surgery; medication and therapy; radiation treatment; combination treatment; nonmedical treatment; adverse effects; opinion, feelings, and experiences regarding providers of health care; cost of health care; and loss of a loved one due to breast cancer.

### Statistical Analysis

Frequency distributions were conducted for the categorical and dichotomous variables and mean with SD and range for continuous variables. The number of views, likes, comments, and shares were summed. We compared differences between TikTok videos that were related to or supported breast cancer advocacy, whether self-advocacy or advocacy on behalf of a loved one, and performed univariable analyses using chi-square test for categorical and dichotomous variables and ANOVA for continuous variables. A random sample of 10% of the videos was coded by a second coder. Discrepancies between the 2 coders were resolved through discussion. The interrater reliability was computed and found to be very high (κ=0.98). All analyses were performed using SPSS statistical software (version 28; IBM Corp) [26]. *P* values <.05 were considered statistically significant.

### Ethical Considerations

This study was exempt from review by William Paterson University’s Institutional Review Board due to the lack of human subject involvement.

## Results

Of the 100 TikTok videos related to breast cancer reviewed, 60% (n=60) were created since 2021 (Table 1). The cumulative number of views of the videos included in this study was 369,504,590. On average, the videos had approximately 3.7 million (SD 3,581,698) views each and collectively were shared more than half a million times. Patients and loved ones of individuals with breast cancer created the greatest number of videos (n=81, 81%). Videos that were related to breast cancer advocacy (n=62, 62%) differed from those that did not (n=38, 38%) by both characteristics and content. Videos advocating for breast cancer received substantially more shares (mean 7396, SD 11,903 vs mean 1290, SD 1951; *P*=.002; Table 1).

The majority (n=83, 83%) of the videos featured an individual who currently has or has had breast cancer, although only 14% (n=14) revealed a new breast cancer diagnosis (Table 2). The most commonly discussed topic was having breast cancer support (n=88, 88%), followed by coping with the myriad issues surrounding breast cancer (n=79, 79%). Between one-third and about one-half of videos talked about important issues such as body image (n=48, 48%), hair loss following treatment (n=38, 38%), surgery (n=46, 46%), medication and therapy (n=41, 41%), adverse effects of treatment (n=33, 33%), or the stigma associated with a breast cancer diagnosis (n=44, 44%). Very few videos addressed radiotherapy (n=1, 1%), combination therapy (n=9, 9%), or nonmedical treatment (n=4, 4%). Advocating videos more often discussed body image (40/62, 64% vs 8/38, 21%; *P*<.001), stigma associated with breast cancer (33/62, 53% vs 11/38, 29%; *P*=.02), and breast cancer surgery (36/62, 58% vs 10/38, 26%; *P*=.002) compared to videos that did not specifically advocate for breast cancer. Among videos with no mentions of breast cancer advocacy, content focused more frequently on near-term breast cancer issues that included hair loss (20/38, 53% vs 18/62, 29%; *P*=.02), anxiety (18/38, 47% vs 9/62, 14%; *P*<.001), coping (35/38, 92% vs 44/62, 71%; *P*=.01), medication and therapy (23/38, 60% vs 18/62, 29%; *P*=.002), and adverse effects of treatment (18/38, 47% vs 15/62, 24%; *P*=.02).

**Table 1:** Differences in the characteristics of TikTok videos (N=100) related to breast cancer among those related to breast cancer advocacy (n=62) and those that did not (n=38).

				Total (N=100)		Advocacy			*P* value	
					Yes (n=62)		No (n=38)			
**Video characteristics**										
	**Year (total: N=100; advocacy, yes: n=62; advocacy, no: n=38), n (%)**									.33
		2019	1 (1)		0 (0)		1 (2.6)			
		2020	14 (14)		9 (14.5)		5 (13.2)			
		2021	60 (60)		35 (56.5)		25 (65.8)			
		January to July 2022	25 (25)		18 (29)		7 (18.4)			
	**Number of views (n=369,504,590)**									
		n (%)	369,504,590 (100)		262,825,700 (71.1)		106,678,890 (28.8)	.05		
		Mean (SD)	3,695,046 (3,581,698)		4,239,124 (3,879,713)		2,807,339 (2,864,258)			
		Range	290,600-18,900,000		290,600-18,900,000		446,000-11,600,000			
	**Number of comments (n=676,604)**									
		n (%)	676,604 (100)		492,062 (72.7)		184,542 (27.3)			
		Mean (SD)	6766 (11,314)		7936 (13,703)		4856 (5183)	.19		
		Range	70-92,500		70-92,500		133-22,000			
	**Number of shares (n=507,638)**									
		n (%)	507,638 (100)		458,603 (90.3)		49,035 (9.7)			
		Mean (SD)	5076 (9879)		7396 (11,903)		1290 (1951)	.002		
		Range	45-66,400		45-66,400		84-11,700			
**Format (total: N=100; advocacy, yes: n=62; advocacy, no: n=38), n (%)**										
	**Uses dance**									.71
		Yes	8 (8)		6 (9.7)		2 (5.3)			
		No	92 (92)		56 (90.3)		36 (94.7)			
	**Uses music**									.13
		Yes	70 (70)		40 (64.5)		30 (78.9)			
		No	30 (30)		22 (35.5)		8 (21.1)			
	**Uses humor**									.13
		Yes	27 (27)		20 (32.3)		7 (18.4)			
		No	73 (73)		42 (67.7)		31 (81.6)			
	**Video creator**									.12
		Patient	68 (68)		42 (67.7)		26 (68.4)			
		Loved one	12 (12)		5 (8.1)		7 (18.4)			
		Health professional	3 (3)		3 (4.8)		0 (0)			
		Company	7 (7)		6 (9.7)		1 (2.6)			
		Consumer	9 (9)		6 (9.7)		3 (7.9)			
		Patient/loved one	1 (1)		0 (0)		1 (2.6)			

**Table 2:** Differences in the content of TikTok videos (N=100) related to breast cancer among those related to breast cancer advocacy (n=62) and those that did not (n=38).

Content		Total (N=100), n (%)	Advocacy			*P* value	
			Yes (n=62), n (%)	No (n=38), n (%)			
**Have or had breast cancer**							.01
	Yes	83 (83)	47 (76)	36 (95)			
	No	17 (17)	15 (24)	2 (5)			
**New breast cancer diagnosis**							<.001
	Yes	14 (14)	3 (5)	11 (29)			
	No	86 (86)	59 (95)	27 (71)			
***BRCA*^a^ genetic mutation**							.37
	Yes	5 (5)	2 (3)	3 (8)			
	No	95 (95)	60 (97)	35 (92)			
**Body image**							<.001
	Yes	48 (48)	40 (64)	8 (21)			
	No	52 (52)	22 (36)	30 (79)			
**Hair loss following treatment**							.02
	Yes	38 (38)	18 (29)	20 (53)			
	No	62 (62)	44 (71)	18 (47)			
**Anxiety**							<.001
	Yes	27 (27)	9 (14)	18 (47)			
	No	73 (73)	53 (86)	20 (53)			
**Stigma associated with breast cancer**							.02
	Yes	44 (44)	33 (53)	11 (29)			
	No	56 (56)	29 (47)	27 (71)			
**Support**							.56
	Having support	88 (88)	56 (90)	32 (84)			
	Lack of support	5 (5)	3 (5)	2 (5)			
	Unknown	7 (7)	3 (5)	4 (11)			
**Coping**							.01
	Yes	79 (79)	44 (71)	35 (92)			
	No	21 (21)	18 (29)	3 (8)			
**Surgery**							.002
	Yes	46 (46)	36 (58)	10 (26)			
	No	54 (54)	26 (42)	28 (74)			
**Medication and therapy**							.002
	Yes	41 (41)	18 (29)	23 (60)			
	No	59 (59)	44 (71)	15 (40)			
**Radiation therapy**							>.99
	Yes	1 (1)	1 (2)	0 (0)			
	No	99 (99)	61 (98)	38 (100)			
**Combination therapy**							>.99
	Yes	9 (9)	6 (10)	3 (8)			
	No	91 (91)	56 (90)	35 (92)			
**Nonmedical treatment**							.63
	Yes	4 (4)	2 (3)	2 (5)			
	No	96 (96)	60 (97)	36 (95)			
**Adverse effects of treatment**							.02
	Yes	33 (33)	15 (24)	18 (47)			
	No	67 (67)	47 (76)	20 (53)			

^a^*BRCA*: breast cancer gene.

## Discussion

To our knowledge, this is the first study describing the content of the most viewed TikTok videos on the larger topic of breast cancer. Our review of the literature revealed one prior publication focusing on fat grafting in breast cancer [27]. Prior research evaluated breast cancer coverage on various social media platforms [16,28]. The findings of this study indicate that the 100 TikTok videos related to breast cancer reviewed were filled with messages created to support and advocate for individuals with breast cancer. Social support has long been noted as being beneficial [29], especially in health situations [30] and more specifically in the case of patients with cancer, survivors, and their loved ones [31-33]. In the digital era, social support can easily take place through technological mediums, whether through preplanned interventions or spontaneously through individual use [22,34-38]. Our findings corroborate with this existing research in that the overwhelming majority of videos in our sample mentioned support, coping, and advocacy [39,40].

Although there is research that indicates the possibility of misinformation spreading on TikTok, as it has on many social media platforms [41,42], emerging research also supports the concept that TikTok can provide a high level of support for those experiencing difficulty [43,44]. This possibility of far-reaching effects of support are enhanced by the widespread reach of TikTok. The nature of expression over social media can lead to greater support as geographic boundaries are eliminated.

Issues affecting the individual both physically and psychologically were frequently noted in the videos we reviewed. The psychological factors that accompany breast cancer include substantial life changes, dealing with a life-threatening illness, and painful treatments. Physical changes and issues related to a new body image also amplify psychological distress [45,46]. Breast cancer surgeries such as biopsies, lumpectomies, mastectomies (total, double, modified radical, radical, nipple-sparing, or skin-sparing), aesthetic flat closures, and breast reconstruction are all options that impact physical appearance [47] and leave many women stigmatized, which can affect quality of life [48]. Video creators discussed the stigma of surgery options that they faced and their experience with chemotherapy. They mentioned not feeling feminine or losing their femininity and hair loss—all part of self-identity and body image. For instance, although hair loss is common with chemotherapy, it can lead to psychological disturbance and stress [49,50].

The limitations of this study include the fact that the design was cross-sectional and that we only reviewed English-language videos. Additionally, only one popular hashtag was used, which can lead to a limited perspective. The relatively small sample of only 100 videos may not represent the full range of videos. Content analysis does not allow an in-depth reflection of how information in these videos is processed and used by viewers. Hence, this is an area for future research. Of note, TikTok is only one social media platform, with content delivered in a specific way. Therefore, findings cannot be generalized across other social media platforms. Further, although patients and loved ones claim to be creating many of the videos included in this study, there is no way to verify this information. However, this study offers perspective into the use of TikTok to discuss breast cancer and the level of support found on this medium.

Social media platforms such as TikTok provide a space for health information to be disseminated to a wide variety of populations with varying health literacy skills [51]. Video creators on this platform used advocacy and support to cope with breast cancer in some respect.

The findings of this study indicate that most of the interest in TikTok videos was around patient journeys, coping mechanisms, and support systems. As cancer interventions have better outcomes in patients with early diagnosis, it is important to reach vulnerable populations at a young age. TikTok, which is predominantly used by young women, represents an ideal platform for outreach by professional societies and advocacy groups focusing on breast cancer. Their campaigns may benefit from incorporating the findings of this study.
